# Early Cytoskeletal Remodeling Drives Hypertrophic Cardiomyopathy Pathogenesis in *MYH6/7* Mutant hiPSC-Derived Cardiomyocytes

**DOI:** 10.3390/jcdd12120500

**Published:** 2025-12-17

**Authors:** Mohammad Shameem, Hassan Salih, Ahmed Sharara, Roshan Nicholas Rochus John, Leo Ogle, Bhairab N. Singh

**Affiliations:** 1 Department of Rehabilitation Medicine, University of Minnesota, Minneapolis, MN 55455, USA; mshameem@umn.edu (M.S.);; 2 Department of Biomedical Engineering, University of Minnesota, Minneapolis, MN 55455, USA; 3 Experimental Surgical Services (ESS), University of Minnesota, Minneapolis, MN 55455, USA; 4 Stem Cell Institute, University of Minnesota, Minneapolis, MN 55455, USA

**Keywords:** hypertrophic cardiomyopathy, MYH6, MYH7, sarcomere, cytoskeletal, hiPSC-CMs, actin

## Abstract

Hypertrophic cardiomyopathy (HCM) is a common and deadly cardiac disease characterized by enlarged myocytes, increased myocardial wall thickening, and fibrosis. A majority of HCM cases are associated with mutations in the β-myosin heavy chain (*MYH7*) converter domain locus, which leads to varied pathophysiological and clinical manifestations. Using base-editing technology, we generated mutant human-induced pluripotent stem cell-derived cardiomyocytes (hiPSC-CMs) harboring HCM-causing myosin converter domain mutations (*MYH7 c.2167C>T [R723C]*; *MYH6 c.2173C>T [R725C]*) to define HCM pathogenesis in vitro. In this study, we integrated transcriptomic analysis with phenotypic and molecular analyses to dissect the HCM disease mechanisms using *MYH6/7* myosin mutants. Our KEGG analysis of bulk RNA-sequencing data revealed significant upregulation of transcripts associated with HCM in the mutant hiPSC-CMs. Further, in-depth transcriptomic analysis using Gene-Ontology (GO-term) analysis for biological process showed upregulation of several transcripts associated with heart development and disease. Notably, our analysis showed robust upregulation of cytoskeletal transcripts, including actin-cytoskeleton networks, sarcomere components, and other structural proteins in the mutant CMs. Furthermore, cellular and nuclear morphological analysis showed that the *MYH6/7* mutation induced cellular hypertrophy and increased aspect ratio compared to the isogenic control. Immunostaining experiments showed marked sarcomere disorganization with lower sarcomeric order and higher dispersion in the mutant hiPSC-CMs, highlighting the remodeling of the myofibril arrangement. Notably, the *MYH6/7* mutant showed reduced cortical F-actin expression and increased central F-actin expression compared to the isogenic control, confirming the cytoskeletal remodeling and sarcomeric organization during HCM pathogenesis. These pathological changes accumulated progressively over time, underscoring the chronic and evolving nature of HCM driven by the *MYH6/7* mutations. Together, our findings provide critical insights into the cellular and molecular underpinnings of *MYH6/7*-mutation-associated disease. These findings offer valuable insights into HCM pathogenesis, aiding in future therapies.

## 1. Introduction

Hypertrophic cardiomyopathy (HCM; OMIM: 192600) is a highly prevalent cardiac disease affecting nearly 1 in 500 people worldwide [1] and is associated with a wide-range of symptoms such as left ventricular outflow obstruction, diastolic dysfunction, severe arrhythmias, fibrosis, and sudden cardiac death (SCD) [2,3]. HCM is an autosomal dominant genetic disease characterized by mutations in sarcomeric protein-encoding genes, including β-myosin heavy chain (β-MHC), myosin binding protein C (MYBPC3), and troponin T2 cardiac type (TNNT2) [4,5]. Mutations in the *MYH7* and *MYBPC3* account for the largest number of HCM cases [6], whereas mutations in troponin C (*TNNC1*), troponin I3 cardiac type (*TNNI3*), and topomyosin1 (*TPM1*) are less common, collectively accounting for fewer than 10% of cases [7]. Additional rare sarcomeric contributors include actin alpha cardiac muscle 1 (*ACTC1*), myosin light chain 2 (*MYL2*) and *MYL3*, and cysteine- and glycine-rich protein 3 (*CSRP3*) [8,9]. Of all the gene mutations associated with HCM, nearly 15–25% are found in the β-myosin heavy chain (*MYH7*; OMIM:160760) gene. Within the *MYH7* locus, the converter domain is considered as a hot-spot for HCM mutation [10]. Mutations in the converter domain are associated with severe HCM phenotypes, including early-onset of disease, increased left ventricular hypertrophy, and higher risk of SCD with highly variably clinical presentation [11]. For example, while R719W leads to severe HCM pathogenesis with increased SCD [12,13], R723G mutation results in altered motor functions and familial HCM [10]. Similarly, G741R results in impaired relaxation kinetics and HCM phenotype [10]. Due to these heterogeneic pathogenesis, the identification of widely applicable therapeutics is not a viable option. We propose that deciphering early changes associated with the converter domain mutation will help define HCM pathogenesis for novel therapies.

Recent advances in genetic engineering and hiPSC-derived cardiomyocytes (hiPSC-CMs) generation has enabled the development of mutation-specific disease models to define the mechanism of disease onset and progression [14,15]. Moreover, hiPSC-CMs provide a pathophysiologically relevant approach to investigate human HCM and to design novel therapeutics [15,16]. Integration of gene editing tools together with iPSC-technology allows for studies of the mutation with an isogenic control to manage the genetic variability of individuals that may influence disease conditions [17]. Recently, Mosqueira et al. demonstrated an HCM phenotype in *MYH7* R453C mutant hiPSC-CMs with sarcomeric disarray and arrhythmia [18]. Similarly, studies using *MYH7* E848G mutant hiPSC-CM showed disrupted interactions between β-MHC and MyBP-C with reduced contractility [19]. These studies support the benefits of hiPSC-CMs to study HCM pathogenesis. Although mutation type alone cannot yet reliably predict disease outcomes or inform clinical management, patients harboring multiple independent sarcomere mutations have been shown to exhibit more severe phenotypes, including an increased incidence of heart failure and SCD [20,21,22]. These observations underscore the importance of comprehensive genetic analysis, particularly in cases involving multiple HCM-associated variants. In this study, we focus specifically on the impact of dual mutations in *MYH6* and *MYH7*.

MYH6 (α-MHC) and MYH7 (β-MHC) proteins are encoded by *MYH6* and *MYH7* genes, respectively [23,24]. MYH6 is the predominant ventricular myosin isoform in small mammals such as mice and rats [25] whereas, in human hearts, its expression becomes largely restricted to the atria, with *MYH7* as the predominant myosin in the ventricular tissue, particularly in mature ventricular cardiomyocytes. Under fetal conditions, however, MYH6 is the predominant ventricular isoform [25]. While MYH6 shares a high degree of sequence homology with *MYH7* [26], they have a varying degree of functions [27]. For example, MYH6 exhibits a higher contractile velocity and approximately 70% greater affinity for actin filament as compared to MYH7 [27,28]. Similar to *MYH7*, multiple mutations in *MYH6* have been linked to HCM [29,30]. Previously, we demonstrated that dual mutations in *MYH6* and *MYH7* (*MYH6/7*) in hiPSC-CMs induce early alterations in extracellular-matrix (ECM) remodeling, integrin expression, and cell–ECM adhesion [5]. Whether this matrix remodeling and outside-in signaling has any implication in cytoskeletal changes is not clear. How the mutations in the myosin locus affect the structural dynamics and the cytoskeleton remodeling is not completely defined during HCM pathogenesis.

Cardiomyocyte differentiation and development is associated with a myriad of changes in their structure and function [31,32,33]. Development of the structural and contractile units are one of the most profound changes that occurs in cardiac lineage [33,34]. How this is impacted in the context of HCM progression is unknown. Although sarcomeric mutation-driven mechanisms in HCM are well documented, cytoskeletal remodeling has only recently been recognized as an important contributor to stress and disease pathogenesis [35,36,37]. Many of these investigations examine mechanical loading [35], as well as microtubule-mediated nuclear constriction [36], in predominantly murine models at isolated time points. How cytoskeletal remodeling unfolds over time and aligns with hypertrophic and nuclear phenotypes remains uncertain in human-relevant *MYH7*-mutant cardiomyocytes. Consequently, longitudinal analyses that track contractile function alongside cytoskeletal and sarcomeric changes are warranted. Defining the early changes in association with HCM pathogenesis will help elucidate novel mechanisms and therapy to delay or prevent HCM pathogenesis using mutant hiPSC-CMs and their isogenic counterparts [5,15,16].

In this study, we sought to expand our previously published work [5] and focus on changes associated with sarcomeric organization and cytoskeleton remodeling. Our previous study showed altered ECM dynamics in the dual *MYH6/7* mutant CMs. In addition to ECM change, whether the myosin-mutations impact cytoskeletal remodeling overtime as HCM progresses is incomplete. Analysis of the previously published bulk RNA-seq datasets [5] revealed transcriptomic changes in the cytoskeleton, contractile machinery, and hypertrophic transcripts expression. Additionally, Fourier transform analysis showed changes in sarcomeric organization and disarray in the mutant CMs. We found changes in actin organization with more central F-actin in the mutant CMs as compared to the control. Finally, our qPCR analysis revealed altered regulation of hypertrophic, cytoskeletal, and cell–cell communication gap junctions. This novel investigation serves as one of the primary studies on elucidating time-based cytoskeletal changes in a disease model, setting the notion for future drug development and disease modeling.

## 2. Materials and Methods

### 2.1. hiPSC Culture and hiPSC-CMs Differentiation

The generation of the hiPSC-*MYH6/7* double mutant lines was previously described [5]. Isogenic hiPSC and hiPSC-*MYH6/7* double mutant cell lines were kindly gifted by Prof Brenda Ogle, Department of Biomedical Engineering at the University of Minnesota, USA. The hiPSC cells were cultured on a matrigel-coated plate using mTeSR1 medium (STEMCELL Technologies, Kent, WA, USA; catalog #85850) at 37 °C in a humidified incubator with 5% CO_2_. Cardiomyocyte differentiation was carried out as previously described [38,39]. Briefly, hiPSCs were washed with 1X DPBS and dissociated into single cells using Accutase solution (Sigma-Aldrich, St. Louis, MO, USA; catalog #A6964). A total of 0.5 × 10^6^ cells were seeded in each well of a matrigel-coated 12-well plate in mTeSR1 medium supplemented with 10 μM of ROCK inhibitor Y-27632 (Selleck Chemicals, Houston, TX, USA; catalog #S1049,). After 24 h, the medium was replaced with fresh mTeSR1 media (2 mL/well). Upon 90–100% confluency (Day 0), the media was replaced with RPMI/B27 minus insulin (Gibco, Thermo Fisher Scientific, Carlsbad, CA, USA; catalog #A18956-01) containing 7 μM CHIR99021 (Sigma-Aldrich, Milwaukee, WI, USA; catalog #SML1046). On day 2 of differentiation (48 h post-CHIR treatment), the media was replaced with RPMI/B27 minus insulin containing 7.5 μM of IWP2 (Tocris Bioscience, Pittsburgh, PA, USA; catalog #3533,). On day 4 of differentiation, the media was replaced with fresh RPMI/B27 minus insulin (2 mL/well). On day 6, the media was replaced with fresh RPMI/B27 containing insulin supplement (RPMI/B27+) (2 mL/well) (Gibco, Thermo Fisher Scientific, Carlsbad, CA, USA; catalog #17-504-044). The media was changed every two days. On day 11, hiPSC-CMs were washed with 1X DPBS and incubated with 0.25% trypsin for 16 min at 37 °C and re-plated at a 1:3 ratio in a matrigel-coated 12-well plate in RPMI/B27+ media supplemented with RI (10 μM). Between days 13–16, cells were washed with 1X DPBS and cultured with no-glucose DMEM (Gibco, Thermo Fisher Scientific, Waltham, MA, USA; catalog #11966025) supplemented with lactate (4 mM) to purify the hiPSC-CM population. On day 16, each well was washed with 1X DPBS, and hiPSC-CMs were maintained in RPMI/B27+ medium with fresh media change every 3 days until experimental use.

### 2.2. RNA-Seq Analysis

Previously published bulk RNA-sequencing data from day 15 hiPSC-CMs (GEO accession GSE198258) were re-analyzed following the approach described in Hsieh et al. [5]. Raw count matrices were processed in DESeq2 using *R*-Studio (4.4.3), using median-of-ratios normalization and a negative binomial model to compute *p*-values and Benjamini–Hochberg-adjusted *p*-values and genes with an adjusted *p*-value < 0.05 were considered differentially expressed. Upregulated genes were defined as those with a log2 fold change greater than 0.8 in *MYH6/7* mutants relative to isogenic controls. Gene symbols were converted to Entrez IDs using the bitr function. Kyoto Encyclopedia of Genes and Genomes (KEGG) pathway enrichment was performed on the upregulated Entrez IDs using enrichKEGG (clusterProfiler) with a *p*-value cutoff of 0.05. Enrichment results were compiled into a data frame (“keggDF”) and used to generate dot plots. From the KEGG results, the pathway “Cytoskeleton in muscle cells (hsa04820)” was selected for further analysis based on their robust dysregulation. Genes associated with this pathway were mapped with bitr function, organized into a data frame comparing *MYH6/7* mutants and controls, converted to a matrix, and visualized using pheatmap. Gene Ontology (GO) enrichment was performed using the enrichGO package focusing on biological process (BP) and molecular function (MF) ontologies. Data frames for each ontology were created from the enrichment output, and dot plots were generated in ggplot2 using the corresponding gene ratio values.

### 2.3. RNA Isolation and qPCR Analysis

RNA isolation and qPCR analysis was performed as described previously [40,41]. Briefly, hiPSC-CMs were lysed using RNA-lysis buffer (Quick RNA kit, Zymo Research, Irvine, CA, USA; catalog #R1057). Total RNA was isolated following the manufacturer’s protocol with the Quick RNA kit, followed by on-column DNA digestion using DNase I to eliminate genomic DNA as per the manufacturer’s instructions. The RNA was eluted with DNase/RNase-free water and quantified by using the Nanodrop instrument. A total of 1 µg RNA was used to synthesize cDNA by using the Superscript IV VILO kit (Thermo Fisher Scientific, Waltham, MA, USA; catalog #11756050) according to the manufacturer’s protocol in a 20 µL reaction volume. Quantitative PCR (qPCR) was performed using gene-specific oligos and the SYBR-green (Applied Biosystems, Foster City, CA, USA, catalog #4367659) method. For the qPCR analysis, each transcript was normalized to the housekeeping gene, EDF1. The list of primers is provided in Supplemental Table S1.

### 2.4. Contractility Analysis

ImageJ-based MYOCYTER software (v1.5) was used to analyze the contractile mechanics of cardiomyocytes as described previously [42]. Contractility videos were collected using the DMi8 Leica Microscope at 20X (Waltham, MA, USA) magnification, with 10 frames per second over 20 s for all videos without pacing. These videos were uploaded into the MYOCYTER program (v1.5) for pretesting. A batch list was created and videos were analyzed at a lower threshold of 0, size of 1600, with a percentage of max recognized as beat being 20% with a detection value at 4. No smoothing, force-reference frame, or manual ROI was selected. The baselined amplitudes and speeds were averaged and plotted. We analyzed 600 to 700 cells for isogenic control and 500 to 600 cells for *MYH6/7* mutant per condition over time. We observed %CV (coefficient of Variation) values consistently above 20% when analyzing beats per minute, contraction times, and relaxation times.

### 2.5. Immunostaining of hiPSC-CMs

Immunostaining was performed as described previously [38,43]. Briefly, hiPSC-CMs cells were washed with 1X DPBS and fixed in the 4% paraformaldehyde solution (Sigma-Aldrich, Milwaukee, WI, USA; catalog #47608) for 15 min at room temperature. For staining, cells were washed with 1X PBS and permeabilized by incubation with 0.2% Triton X-100 (Sigma-Aldrich, Milwaukee, WI, USA; catalog #X100) for 45 min at room temperature. After permeabilization, cells were washed twice with 1X PBS and blocked with BGST (50 g/L BSA: Sigma-Aldrich; Milwaukee, WI, USA; catalog #A9418, 10 g/L glycine: Sigma-Aldrich; Milwaukee, WI, USA; catalog #50046, 2% Donkey Serum: Abcam; Waltham, MA, USA; catalog #ab7475, 0.1% Triton-X-100) solution for 2 h at room temperature. Cells were incubated with primary antibody α-actinin (1:500; Abcam; Waltham, MA, USA; catalog #ab9465) diluted in BGST solution overnight at 4 °C. After the overnight incubation, the cells were washed twice with 0.2% Tween-20-PBS and once with 1X PBS. After that, cells were incubated with secondary antibodies Alexa Fluor 488 (1:400; Thermo Fisher Scientific, Carlsbad, CA, USA; catalog #A21202) for 1.5 h and then washed twice with 0.2% Tween-20-PBS and once with 1X PBS. Cells were then stained with rhodamine phalloidin fluorescent dye (1:1000; Abcam; Waltham, MA, USA; catalog #AB235138) to label filamentous-actin (F-actin) diluted in BGST solution for 30 min at RT. Cells were washed twice with 0.2% Tween-20-PBS and once with 1X PBS. After that, cells were incubated with 1 µg/µL DAPI diluted in 1X PBS for 10 min and washed with 1X PBS three times. Stained cells were imaged at 40X magnification using an EVOS M5000 fluorescent microscope (Thermo Fisher Scientific, Waltham, MA, USA).

### 2.6. hiPSC-CMs Cell Size Analysis

To quantify hiPSC-CMs cell size, we utilized the measurement function on ImageJ to determine cell area and perimeter [38]. Brightfield images were captured at day 20, day 30, and day 45 using the DMi8 Leica Microscope (Waltham, MA, USA) at 20X magnification. Within ImageJ, we used the “Set Measurements” option within the “Analyze” tab to select “Area” and “Perimeter” measurement options. The individual cells were outlined manually using the lasso function. Once a cell was selected, using ‘Ctrl-M’ would catalog the values. For each point, ≥6 images were analyzed with a minimum of ≥30 cells per image analyzed. Quantification of images was performed by an expert in a blinded fashion. Images were assigned random codes, and the analysis expert was not aware of the sample codes. The codes were then matched after the completion of the analysis.

### 2.7. MorphoScript Analysis

MorphoScript analysis was performed as described previously by Homan et al. 2021 [44]. The MATLAB-based program (R2024b) was used to quantify sarcomere order and dispersion using α-actinin-stained hiPSC-CMs images, obtained from the above-described method (Section 2.5), captured at 40X magnification using an M5000 EVOS microscope (Thermo Fisher Scientific, Waltham, MA, USA). Initially, DAPI-stained images were used to identify individual cells and distinguish them from clusters by outlining their boundaries. MATLAB post-processing was used to enhance the contrast of the images by 0.35% and then uploaded into MATLAB with a pixel-to-micron conversion factor of 0.227 µm. The analysis of sarcomere order and dispersion was performed automatically using the model with a window size of 15, an overlap of 0.5, and a detection threshold of 0.0065. If sarcomere orientation did not match the alignment on the TIF image, the “edit” function was used to correct the Fourier-based sarcomere alignment as seen in the image. To optimize the detection parameters, the threshold value was averaged from three cells showing clear striations and applied consistently across all cells analyzed.

### 2.8. Statistical Analysis

Statistical significance and *p*-value between the isogenic control and the *MYH6/7* mutant was assessed using an unpaired Welch’s *t*-test or Mann–Whitney U test as appropriate. Analyzed data are presented as mean ± SD. Error bars represent the standard deviation (SD). All statistical analyses were conducted using GraphPad Prism version 10.6.1 (GraphPad Software, La Jolla, CA, USA).

## 3. Results

### 3.1. MYH6/7 Mutations Induce Differential Expressions of Cytoskeleton-Related Transcripts

**Figure 1:**
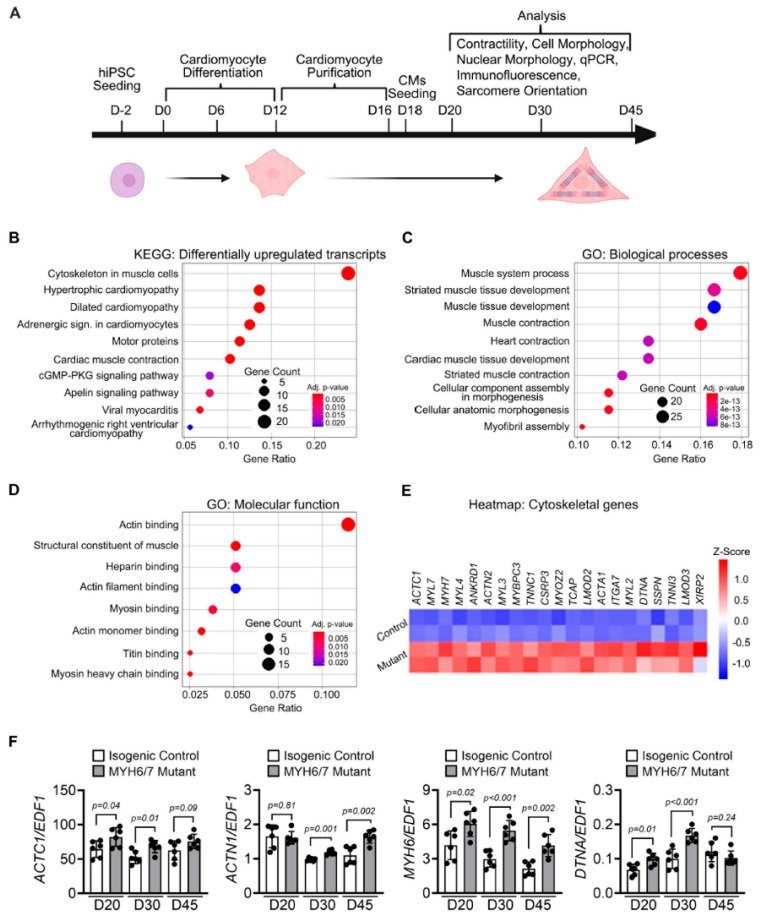
*MYH6/7* mutations upregulate the cytoskeletal transcripts in hiPSC-CMs. (**A**) Schematic illustrating the stepwise differentiation of hiPSC-CMs and the experimental timeline used for cellular and molecular analyses (figure generated using BioRender.com). (**B**) KEGG pathway analysis using the upregulated transcripts from bulk RNA-sequencing data highlights significant upregulation of cardiomyopathy-related genes. (**C**) GO-term analysis for biological process showed enrichment of cytoskeleton-associated processes. (**D**) GO-term analysis for molecular function showed the increased expression of genes involved in structural and cytoskeletal functions. (**E**) Heatmap displaying upregulated cytoskeletal genes in *MYH6/7* mutant hiPSC-CMs compared to controls. (**F**) Validation of the bulk RNA-seq analysis by qPCR. Expression levels of cytoskeletal transcripts (*ACTC1*, *ACTN1*, *MYH6*, and *DTNA*) were analyzed by qPCR at day 20, day 30, and day 45. (n = 6) (Each dot represents an individual technical replicate from *n* = 3 independent biological replicates). Data are presented as mean ± SD. Statistical significance and the *p*-value between the isogenic control and *MYH6/7* mutant was determined by using unpaired Welch’s *t*-test.

To elucidate the molecular and cellular impact of *MYH6/7* mutations (Supplemental Figure S1A–C) during the different stages of HCM progression (as illustrated in Figure 1A), we utilized our previously published bulk RNA-seq analysis on isogenic control and mutant hiPSC-CMs [5]. Our previous study revealed that the *MYH6/7* mutant hiPSC-CMs exhibit early disruptions in ECM remodeling [5]; but, whether this outside-in signaling contributes to structural remodeling in HCM is unclear. To identify differentially upregulated transcripts, we performed differential gene expression analysis and identified a distinct transcriptional profile in the *MYH6/7* mutant. KEGG pathway analysis revealed a significant enrichment of cytoskeleton-related pathways in the *MYH6/7* mutant (Figure 1B). We observed robust upregulation in cytoskeletal transcripts, indicating significant changes in contractile dynamics and sarcomeric unit disruption. Furthermore, our GO enrichment analysis showed significantly enriched biological processes related to the muscle system, cell morphology, muscle organization, and contractile process (Figure 1C). To further dissect the molecular basis of contractile dysfunction, we analyzed GO terms related to molecular function. Among the most enriched functions, actin binding functions exhibited the highest level of upregulation, encompassing 18 actin dynamics-related transcripts and accounting for 12.5% of the total gene count (*p* < 0.05) (Figure 1D). To visualize these transcriptional changes, we generated a heatmap highlighting the upregulation of contractile and structural transcripts associated with cytoskeletal organization, including *ACTC1*, *MYL2*, *MYH7*, *ACTN2*, *MYBPC3*, and *TNNI3* (Figure 1E). These findings highlighted that altered contractile dynamics are prevalent in the *MYH6/7* mutant. To validate these RNA-seq findings, we performed qPCR analysis to determine the transcript expression profile using hiPSC-CMs. Our results confirmed the significant upregulation of *ACTC1*, *ACTN1*, *MYH6*, and *DTNA* transcripts in the *MYH6/7* mutant compared to isogenic control hiPSC-CMs between days 20–45 (day 20–45) of differentiation (Figure 1F). Further, our data showed increased expression of *MYH7* in the mutant cardiomyocytes relative to the control cardiomyocytes overtime (Supplemental Figure S1D). These findings reveal a strong association between *MYH6/7* mutations and early transcriptional reprogramming of cytoskeletal and contractile genes, supporting cytoskeletal remodeling as a key driver of cardiac dysfunction during HCM progression.

### 3.2. Contractility Assessment in MYH6/7 Mutant hiPSC-CMs

**Figure 2:**
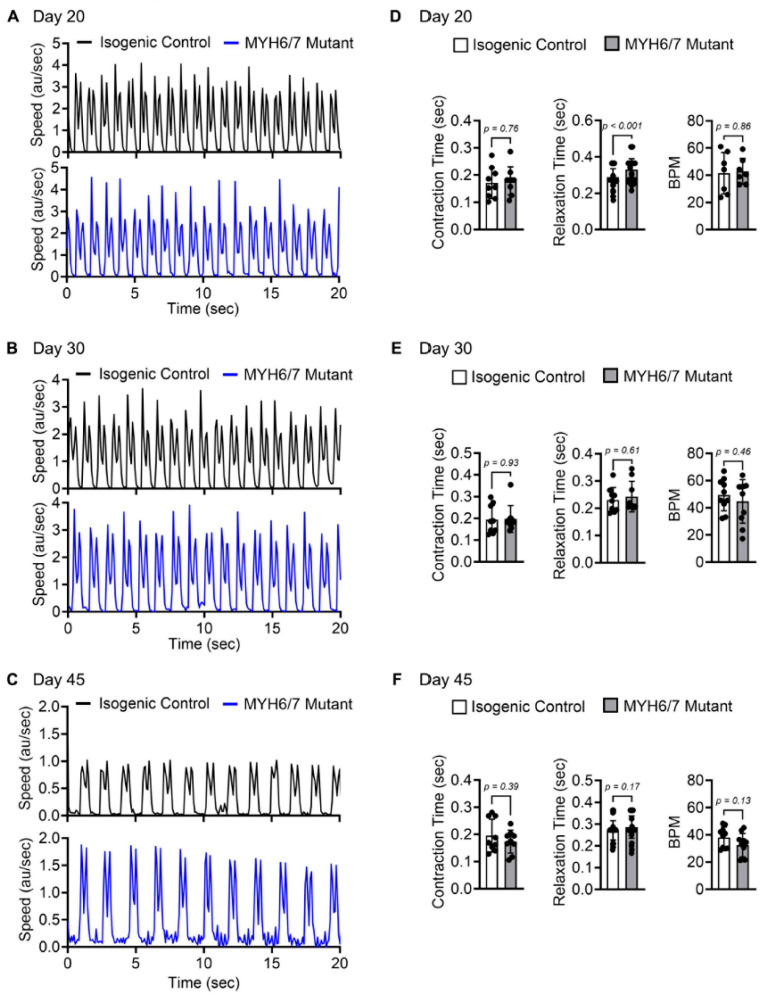
Contractile function assessment in the *MYH6/7* mutant hiPSC-CMs. Representative contractile traces recorded at day 20 (**A**), day 30 (**B**), and day 45 (**C**) showed contractility behavior in *MYH6/7* mutant (blue) compared to isogenic control (black). (**D**–**F**) Contraction time, relaxation time, and BPM (beats per minute) were quantified at day 20 (**D**), day 30 (**E**), and day 45 (**F**) for isogenic control and *MYH6/7* mutant (n = 9) (Each dot represents a technical replicate from n = 3 independent biological replicates). Data are presented as mean ± SD. Statistical significance and the *p*-value between the isogenic control and *MYH6/7* mutant was determined by using unpaired Welch’s *t*-test.

To measure the contractile functions, we differentiated isogenic control and *MYH6/7* mutant hiPSC using our established protocol to hiPSC-CMs and cultured over a 45-day period [5,38]. Contractility videos were collected using the DMi8 Leica Microscope at 20X magnification, with 10 frames per second over 20 s without pacing. Contractility analyses were performed on days 20, 30, and 45, following 48 h of CMs replating (as illustrated in Figure 1A). Mutations in sarcomeric proteins typically impair contractile functions in HCM, with altered force generation and contraction/relaxation cycles being among the major hallmarks [45,46]. To investigate how cytoskeletal abnormalities affected contractile dynamics in the *MYH6/7* mutant, we used the vector-based tool, MYOCYTER, to analyze contractility parameters over time [42]. We found that the *MYH6/7* mutant hiPSC-CM showed a significant impairment in relaxation time at day 20 (0.287 ± 0.04 s in control vs. 0.328 ± 0.06 s in the *MYH6/7* mutant CM; *p*-value = 0.001) (Figure 2A–D). Notably, we did not find any significant difference in the contraction or relaxation times at day 30 and day 45 of differentiation between control and *MYH6/7* mutant (Figure 2E,F). Furthermore, beats per minute (BPM) showed a reducing trend at both day 30 (49.4 ± 11.6 in control vs. 44.7 ± 16.0 in the *MYH6/7* mutant CM; *p*-value = 0.46) and day 45 (38.1 ± 7.6 in control vs. 32.5 ± 8.5 in the *MYH6/7* mutant CM; *p*-value = 0.13) of differentiation; however, this was not significant (Figure 2E,F). We did not observe any significant change in the max amplitude and peak time at the indicated time period between control and *MYH6/7* mutant CMs (Supplemental Figure S2A,B). An increase in relaxation times at the early stage (day 20) indicated relaxation impairment, reflecting functional instability in the mutant and further highlighting contractile dysfunction.

### 3.3. MYH6/7 Mutations Alter Cellular and Nuclear Morphology in hiPSC-CMs

**Figure 3:**
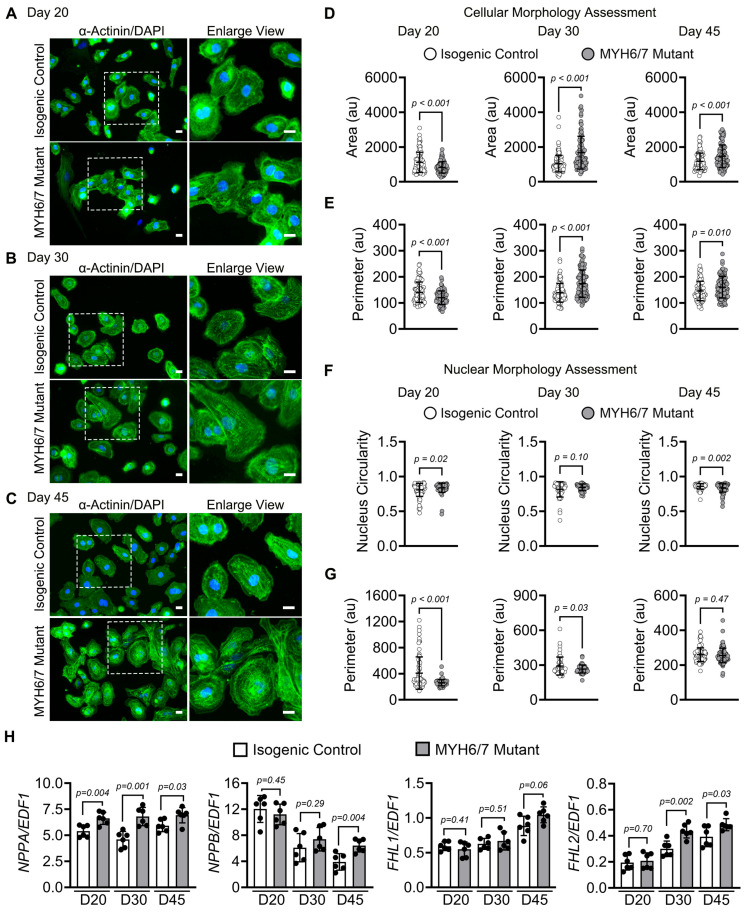
*MYH6/7* mutations induce cellular hypertrophy and nuclear abnormalities in hiPSC-CMs. (**A**–**C**) Representative immunofluorescence images of hiPSC-CMs stained for α-actinin (green) and DAPI (blue), showing the increased cell size in *MYH6/7* mutant hiPSC-CMs compared to isogenic control at day 20 (**A**), day 30 (**B**), and at day 45 (**C**) Scale bar: 50 μm. (**D**,**E**) Brightfield images were used to quantify hiPSC-CMs cell area (**D**) and cell perimeter (**E**), revealing a significant increase in *MYH6/7* mutant compared to isogenic control over time. Analysis was performed using ~200–300 hiPSC-CMs per condition, obtained from *n* = 3 independent biological replicates. Data are presented as mean ± SD. Statistical significance and the *p*-value between the isogenic control and *MYH6/7* mutant were determined using unpaired Welch’s *t*-test. (**F**,**G**) Nuclear morphology analysis indicates a decrease in nuclear circularity (**F**) and an increase in nuclear perimeter (**G**) in the *MYH6/7* mutant compared to isogenic control over time. A circularity index value of 1 represents a perfect circle. Deviation from 1 indicates nuclear shape abnormalities in the *MYH6/7* mutant compared to isogenic control. Analysis was performed using ~150–300 hiPSC-CMs per condition, obtained from *n* = 3 independent biological replicates. Nuclei were stained with DAPI (blue). Data are presented as mean ± SD. Statistical significance and the *p*-value between the isogenic control and *MYH6/7* mutant were determined using unpaired Welch’s *t*-test. (**H**) Relative expression of hypertrophic marker genes (*NPPA*, *NPPB*, *FHL1* and *FHL2*) were analyzed by qPCR at day 20, day 30, and day 45, demonstrating transcriptional upregulation in *MYH6/7* mutants compared to isogenic control. *n* = 6 (Each dot represents an individual technical replicate from three independent biological replicates in duplicates). Data are presented as mean ± SD. Statistical significance and the *p*-value between the isogenic control and *MYH6/7* mutant were determined by using unpaired Welch’s *t*-test.

HCM is characterized by cellular hypertrophy and altered structural dynamics [47,48]. Therefore, we investigated whether the *MYH6/7* mutation affects cellular and nuclear morphology. We performed immunostaining using α-actinin antibodies and imaging using fluorescence microscopy to visualize them. Nuclei were stained with DAPI. These α-actinin-stained hiPSC-CMs showed an increase in the size in the *MYH6/7* mutant at day 30 and day 45 of differentiation as compared to the isogenic control (Figure 3A–C). Notably, it was apparent that the mutant CM showed a sudden morphological change from day 20 onward with increased size (Figure 3A–C). We also observed that the hypertrophy phenotype in the *MYH6/7* mutant hiPSC-CMs was highly variable, indicating the degree of heterogeneity (similar to heterogenic phenotype in HCM patient). Further quantitative assessment of cell area showed that *MYH6/7* mutant CMs exhibit a significant increase in cell area and perimeter compared to isogenic control at both day 30 and day 45 (Figure 3D,E). Next, we evaluated the nuclear architecture at these time points. In general, healthy cells tightly regulate nuclear dimensions, and deviations in nuclear morphology (shape or size) are associated with various pathological conditions [49]. We analyzed the DAPI-stained images of the *MYH6/7* mutant and the isogenic control to measure the nuclear circularity index, perimeter, and aspect ratio. We found that on day 20, the *MYH6/7* mutant CMs showed significantly higher circularity index compared to the isogenic control (Figure 3F), indicative of more rounded nuclei. However, by day 45, the nuclear circularity was significantly reduced (Figure 3F), suggesting progressive nuclear remodeling and deformation in the *MYH6/7* mutants. Additionally, the nuclear perimeter significantly decreased both on day 20 and day 30 with non-significant change in *MYH6/7* mutants at day 45 compared to the isogenic control (Figure 3G). Further assessment of the nuclear area indicated no significant difference between control and *MYH6/7* mutants over time. However, the nuclear aspect ratio was increased significantly (*p*-value = 0.01) at day 45 in the *MYH6/7* mutant compared to isogenic control (Supplemental Figure S3A,B). Further, we analyzed the frequency distribution of cell nuclear areas and found that no significant differences were observed between the isogenic control and *MYH6/7* mutant on day 20 (*p*-value = 0.55), day 30 (*p*-value = 0.22), or day 45 (*p*-value = 0.44) (Supplemental Figure S3A–E). To further validate these morphological changes, we performed qPCR analysis of cellular hypertrophy. We observed a significant upregulation of *NPPA*, *NPPB*, and *FHL2* at day 45, with no significant difference in the *FHL1* level in the *MYH6/7* mutant compared to the isogenic control (Figure 3H), confirming a hypertrophic phenotype of the *MYH6/7* mutant. Further, we observed significantly reduced expression of *RYR2* in the *MYH6/7* mutant at day 45, whereas the expression of *GJA1* and *KCNJ2* transcripts remained unchanged across different time points when compared to the isogenic control (Supplemental Figure S4A–C). These findings suggest that the *MYH6/7* mutation induces molecular, nuclear, and cellular changes, which leads to HCM progression.

### 3.4. MYH6/7 Mutations Induce Disorganized Sarcomeres in hiPSC-CMs

**Figure 4:**
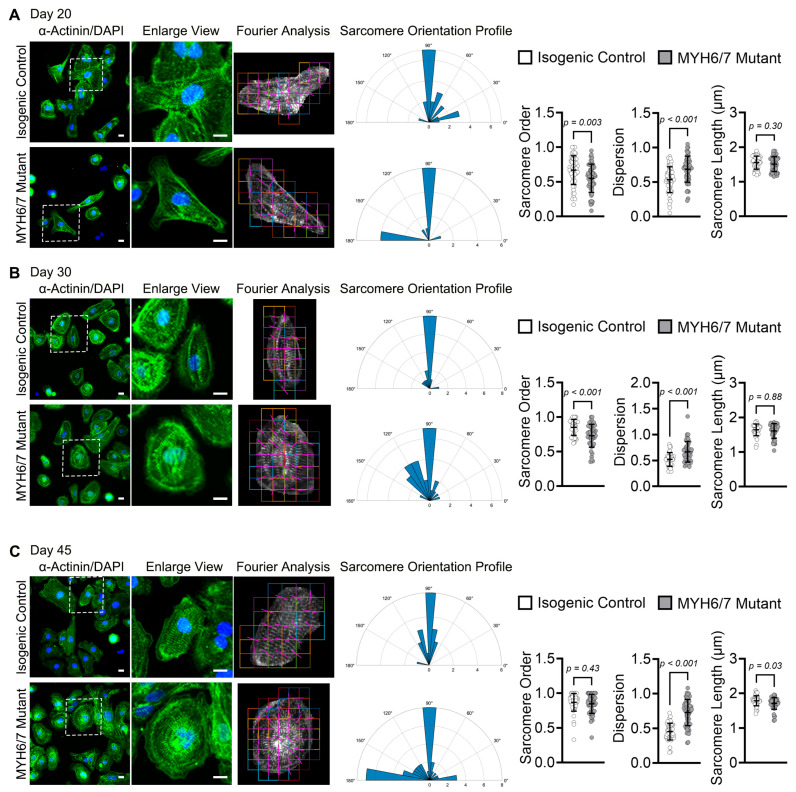
*MYH6/7* mutations alter sarcomere organization in hiPSC-CMs. (**A**–**C**) Representative immunofluorescence images of hiPSC-CMs stained for α-actinin (green), used for quantitative MorphoScript analysis of sarcomere arrangement. These analyses utilized images obtained from immunostaining experiment for morphological assessment (Figure 3). Sarcomere orientation, dispersion, and sarcomere length were quantified at day 20 (**A**), day 30 (**B**), and day 45 (**C**), revealing significant disorganization of sarcomere structure in *MYH6/7* mutants compared to isogenic control. A total of 60–100 cells were analyzed per group. Each data point represents an individual technical replicate from *n* = 3 independent biological replicates. Nuclei were stained with DAPI (blue). Scale bar: 50 μm. Data are presented as mean ± SD. Statistical significance and the *p*-value between the isogenic control and *MYH6/7* mutant were determined using unpaired Mann–Whitney U test.

HCM is characterized by disorganized sarcomere architecture, which compromises force transmission, disrupts contractile function, and contributes to the progression of cardiac hypertrophy [50]. Disruption in sarcomere integrity or derangement directly impairs the mechanical performance of cardiomyocytes [51]. To determine whether the *MYH6/7* mutation alters the sarcomere integrity and arrangement. We performed fluorescence microscopy using control and mutant CMs stained with α-actinin antibodies, and performed MorphoScript analysis using a MATLAB-based program (R2024b) to quantify the sarcomere alignment over time. Our results demonstrated that the *MYH6/7* mutation significantly reduced the sarcomere alignment (ordered sarcomere) compared to the isogenic control (Figure 4A–C). On the other hand, quantification of the sarcomere dispersion showed significantly increased value in the *MYH6/7* mutant compared to the isogenic control over time (Figure 4A–C). It is important to note that while we found highly dispersed sarcomeres in control CMs, sarcomeres displayed ordered and high degree of organized features over a 45-day period, indicative of normal developmental maturation (Figure 4A–C). In contrast, the degree of dispersion was more profound in the mutant CMs (Figure 4A) and consistently displayed a higher degree of sarcomeric disorganization at all time points (Figure 4A–C). Interestingly, sarcomere length remained unchanged at early time points (day 20–30) (Figure 4A,B) but showed a significant reduction at the later time points (day 45) in the *MYH6/7* mutant relative to the isogenic control (Figure 4C). Overall, our results suggest that reduction in the ordered sarcomere and increase in the dispersion showed structural remodeling in the *MYH6/7* mutant, contributing to impaired contractile function during HCM progression.

### 3.5. MYH6/7 Mutations Alter F-Actin Dynamics in hiPSC-CMs

**Figure 5:**
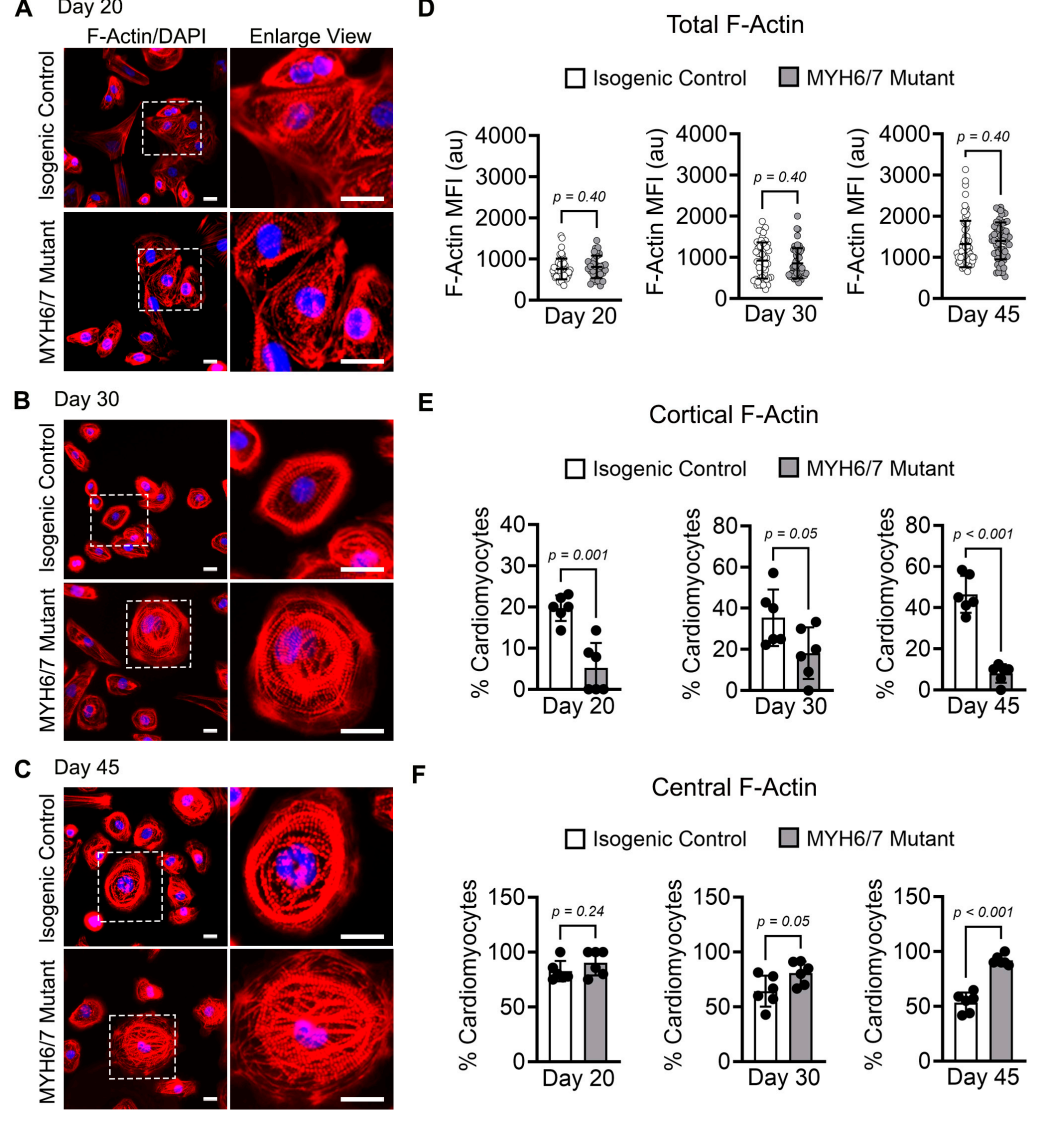
*MYH6/7* mutations induce progressive F-Actin remodeling in vitro. (**A**–**C**) Representative immunofluorescence images of hiPSC-CMs stained for F-actin (Red) and DAPI (nuclei, blue), comparing *MYH6/7* mutant and isogenic control at day 20 (**A**), day 30 (**B**), and day 45 (**C**). Scale bar: 50 μm. The boxed region is magnified in the right panel. (**D**) Quantification of total F-actin mean fluorescence intensity (MFI) shows no significant difference between *MYH6/7* mutant and isogenic control over time. A total of 50–60 cells were analyzed for each group obtained from *n* = 3 independent biological replicates. (**E**) Quantitative analysis of percentage of CMs with cortical F-actin expression, decreases over time in the *MYH6/7* mutant compared to isogenic cells. A total of 50–60 cells were analyzed for each group obtained from *n* = 3 independent biological replicates. (**F**) Quantitative analysis of percentage of CMs with high central F-actin expression increases over time in the *MYH6/7* mutant compared to isogenic cells. A total of 50–60 cells were analyzed for each group obtained from *n* = 3 independent biological replicates. Data are presented as mean ± SD. Statistical significance and the *p*-value between the isogenic control and *MYH6/7* mutant were determined using unpaired Welch’s *t*-test.

Filamentous-actin (F-actin) is the key structural component of the cytoskeleton, assembly and acts as a crucial link between the sarcomere and the cytoskeletal network [52,53]. It plays an important role in maintaining cytoskeletal integrity and contractile mechanics [33,53]. Given its significance and our data indicating changes in cytoskeleton components as a major transcriptome change (Figure 1), we investigated whether the *MYH6/7* mutation impacts F-actin dynamics, potentially leading to early cytoskeletal disorganization. To evaluate this, we performed fluorescence staining to analyze F-actin expression and distribution in the control and *MYH6/7* mutant hiPSC-CMs between day 20–45 of differentiation (Figure 5A–C). We found no significant difference in total F-actin expression between *MYH6/7* mutants and isogenic controls at any time points, indicating that the F-actin turnover was not changed during these time periods (Figure 5D). Interestingly, our analysis showed a significant reduction in cortical F-actin (peripheral F-actin) in *MYH6/7* mutant hiPSC-CMs over time compared to isogenic controls (Figure 5E), indicating cytoskeletal remodeling during HCM progression. Conversely, central F-actin localization increased significantly in *MYH6/7* mutant hiPSC-CMs over time compared to isogenic control (Figure 5F) at day 30 and day 45 of differentiation. These findings suggest that the *MYH6/7* mutation leads to aberrant F-actin organization, characterized by reduced cortical anchoring and increased central localization. This impaired cytoskeletal architecture contributes to the observed contractile dysfunction in the *MYH6/7* mutant hiPSC-CMs, highlighting a novel potential mechanistic link between cytoskeletal remodeling and HCM pathogenesis.

## 4. Discussion

In the present study, we characterize sarcomere organization, contractile dynamics, gene expression, and actin remodeling associated with the *MYH6/7* mutation over time using the hiPSC-CMs model system. During a 45-day differentiation time period, we observed progressive maladaptive changes in mutant cardiomyocytes. Mutant hiPSC-CMs exhibited characteristics of HCM with increased size and structural abnormalities. Quantitative analysis of sarcomeric organization revealed significant disarray or minimal structural definition, supporting the observed dysfunction and confirming the impact of the *MYH6/7* mutation in driving early cytoskeletal remodeling and architectural disruption in cardiac cells.

Using our bulk RNA-seq, KEGG enrichment analysis revealed coordinated upregulation of cytoskeletal remodeling and hypertrophic signaling pathways, consistent with previously published results showing genes such as *MYBPC3*, *ACTN2*, and *TNNT2* to be significantly upregulated [54]. These results suggest that dysregulation of cytoskeletal transcripts represents the major molecular change underlying pathological phenotypes in HCM. Transcripts related to cardiac muscle contraction showed greater evidence for the dysregulation of myofibrillar markers, including *MYH7*, *MYL2*, *MYL4*, *TNNI3*, *ACTA1*, and *ACTC1*, hence providing evidence of cytoskeletal disruption in HCM. Furthermore, our qPCR analysis showed increased expression of *MYH6* levels in the mutant CMs. We predict that this increased expression is mainly to compensate the contractile dysfunction in the *MYH7-homozygous* mutant CMs. Of note, our bulk RNA-seq data showed high levels of *MYH7* in mutant CMs. Previously, we found a modest but non-significant increase in *MYH7/MYH6* ratio [5], indicating that both *MYH6* and *MYH7* were increased in the mutant CMs. Supporting this, studies focused on another distinct point mutation at the 723 positions; *MYH7*-R723G showed highly stable mutant transcripts and allelic imbalance with ~67% contribution of the mutant allele than the wild-type allele [55,56]. In contrast, other *MYH7* mutations including I736T, R719W, and V606M showed reduced mutant *MYH7* mRNA fractions [56]. Indeed, our results showed increased expression of *MYH7* in mutant cardiomyocytes relative to the control. Based on these published data and our findings, we predict that *mutation-locus* dictate the *MYH7* transcripts levels, with mutation at the 723-position has a higher mRNA stability and expression, and the mutant CMs attempt to re-balance the MYH6 and MYH7. Additional studies are needed to explore these changes in the myosin levels in a mutation-specific manner.

In this study, we focused on transcripts related to cytoskeletal organization and remodeling. Our analysis revealed that the levels of *ACTC1* transcript increased significantly between day 20–30 with no significant changes in its levels at day 45. On the other hand, the expression of *ACTN1* remained unchanged at early time points and only increased significantly at a later time point. Both of these molecules are involved in myofibril assembly and crosslinking cytoskeletal filaments. These data support the notion that while the overall filamentous cytoskeleton is not altered, the rearrangement of myofibrillar structure is mediated via interplay between *ACTC1-ACTN1* expression, resulting in cytoskeleton remodeling. This altered F-actin distribution contributes to the contractile and cytoskeletal defects observed in *MYH6/7* mutants compared with isogenic controls. These findings are further supported by our imaging and sarcomeric quantification, reinforcing a model of stress-induced cytoskeleton remodeling in HCM. We predict that cytoskeletal changes, together with cross-talk between cardiomyocyte and cardiac fibroblast, might lead to heightened pathogenic response [57]. In support of this, several studies have indicated adverse cardiac remodeling mediated via activated fibroblast. Co-culture experiments and/or engineered tissue experiments would be needed to address these outstanding questions for the myosin mutants [14].

We found that, over time, contractile dynamics in the mutant condition displayed slightly lower beating. Related observation is also referenced by a study hypothesizing reduced contractile stress as a prevalent anomaly in HCM [45]. We found significantly dysregulated relaxation time on day 20, implying initial sarcomeric remodeling, as evidenced by the disparity in sarcomere dispersion values on day 30 and day 45. Although contraction and relaxation times did not change overtime, we observed a decreasing trend in BPM on day 45 that corresponded to an elevation of sarcomeric dispersion in the *MYH6/7* mutant. Based on these data, we predict that the disorganized sarcomere structure disrupts the alignment and coordination of contractile units, leading to impaired force generation and overall contractile dysfunction in cardiomyocytes, as observed in HCM [50].

Next, our analysis showed that the *MYH6/7* mutation led to cellular hypertrophy with increase in cell size and area. These morphological changes are consistent with patient reports indicating a larger cell size to correspond with myocardial infarction and progression of heart disease [58]. Also, high stiffness (tissue culture plates) prompts cardiomyocytes to enlarge by adding more sarcomeres, allowing them to meet the heightened functional demand in HCM [59]. Notably, we found that *MYH6/7* mutation not only alters cellular morphology but also induced changes in nuclear morphology. Our analysis showed that *MYH6/7* mutation alters the nuclear morphology by altering the circularity and size of the nucleus compared to the isogenic control, confirming the alteration of nuclear structure and genome organization in cardiomyocytes that was observed in HCM [60]. A recent study showed that the cardiomyopathy-associated desmin mutation, p.R127P, severely disrupts filament assembly, compromising the structural integrity of cardiomyocyte and contributing to increased cardiac morbidity and mortality [61]. In a similar vein, our data showed that the *MYH6/7* mutant hiPSC-CMs exhibited reduced cortical F-actin and increased central F-actin localization compared to the isogenic control, suggestive of impaired cytoskeleton dynamics. This redistribution of F-actin likely alters nuclear–cytoskeletal coupling and mechanical force transmission to the nucleus and leads to abnormal nuclear morphology in HCM [60,62,63]. However, the mechanism of remodeling cytoskeleton elements is not clear from our study. It is possible that high stiffness and mechanical overload in the *MYH-mutants* leads to activation of the RhoA-ROCK pathway as these pathways are shown to be involved in actin-cytoskeleton remodeling in HCM pathogenesis [64,65]. In fact, our bulk RNA-seq data showed increased expression of transcripts involved in myofibril assembly, actin filament binding, etc. Based on these data and published findings, we propose that the observed redistribution is possibly mediated via RhoA-ROCK activation, leading to F-actin polymerization and redistribution. Indeed, several studies have shown that the inhibition of Rho kinase signaling reduces cardiac hypertrophy [66,67]. However, involvement of other factors cannot be ruled out in mediating this process. Future studies are necessary to further define the mechanism of cytoskeleton remodeling in these mutant CMs.

**Figure 6:**
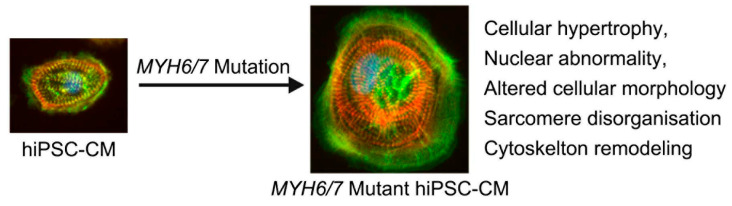
Cytoskeletal remodeling mediates HCM pathogenesis. The schematic illustrates the effects of *MYH6/7* mutations on hiPSC-CMs, highlighting key pathological changes. These mutations lead to cellular hypertrophy, nuclear abnormalities, sarcomere disorganization, and cytoskeletal remodeling, collectively disrupting the normal structure and function of cardiomyocytes. By undertaking in-depth analysis, we found that changes in the cytoskeletal dynamics are associated with early pathogenesis, which then transpire to the surrounding environment to activate the subsequent downstream process. We propose that targeting these early cytoskeletal changes might provide novel therapeutics to prevent or delay HCM progression.

Overall, our study characterized disease progression in *MYH6/7* mutant hiPSC-CMs over a 45-day period, revealing progressive sarcomeric disorganization and cytoskeletal remodeling. Bulk RNA-seq and qPCR analyses showed significant upregulation of genes related to hypertrophy, cytoskeletal, and contractile machinery, while Fourier transform analysis quantified increasing sarcomere disarray (Figure 6). These findings establish a time-resolved in vitro model for HCM pathogenesis, offering valuable insights for future therapeutic development.

## 5. Conclusions

In the present study, we demonstrated that *MYH6/7* mutant hiPSC-CMs showed significant upregulation of cytoskeletal and sarcomeric transcripts, along with disorganized sarcomere structure. Functionally, these cells showed impaired contractility, characterized by reduced beat rate and increased relaxation time. Alterations in the cellular, nuclear morphology and localization of F-actin confirm the impaired cytoskeleton remodeling. These molecular and functional abnormalities progressively worsened over time, highlighting the time-dependent nature of *MYH6/7* mutation-associated HCM pathogenesis. Overall, our study provides novel insights related to early changes associated with *MYH6/7* mutation-induced HCM for future therapeutic strategies. Testing new drugs to modulate the cytoskeletal changes to prevent or delay HCM progression will help in drug discovery and treatment.

## Data Availability

The original contributions presented in this study are included in the article/Supplementary Material. Further inquiries can be directed to the corresponding author.

## Supplementary Materials

The following supporting information can be downloaded at: https://www.mdpi.com/article/10.3390/jcdd12120500/s1, Figure S1: DNA sequencing chromatogram for base-edited hiPSC cells and MYH7 expression analysis; Figure S2: Contractility assessment in *MYH6/7* mutant CMs; Figure S3: *MYH6/7* mutations alter nuclear morphology in hiPSC-CMs; Figure S4: qPCR analysis of Ca^2+^ handling transcripts in control and mutant hiPSC-CMs; Table S1: List of qPCR primers used in this study; Table S2: List of antibodies and staining reagents used in this study.
