# Supplementary material for: Early Cytoskeletal Remodeling Drives Hypertrophic Cardiomyopathy Pathogenesis in MYH6/7 Mutant hiPSC-Derived Cardiomyocytes
Source: J Cardiovasc Dev Dis. 2025 Dec 17;12(12):500. doi: 10.3390/jcdd12120500 (PMC12734156; doi:10.3390/jcdd12120500)
Supplement: Supplementary file 1 [file jcdd-12-00500-s001.zip › jcdd-3964183-supplementary.pdf]

Early Cytoskeletal Remodeling Drives Hypertrophic Cardiomyopathy Pathogenesis in *MYH6/7* Mutant hiPSC-derived Cardiomyocytes

Mohammad Shameem[1#], Hassan Salih[1, 2#], Ahmed Sharara[1, 2], Roshan Nicholas Rochus John[1, 2], Leo Ogle[3], Bhairab N. Singh[1, 4*]

[1]Department of Rehabilitation Medicine, University of Minnesota, MN, USA
[2]Department of Biomedical Engineering, University of Minnesota, MN, USA
[3]Experimental Surgical Services (ESS), University of Minnesota, MN, USA
[4]Stem Cell Institute, University of Minnesota, MN, USA

Supplemental Figure S1. DNA sequencing chromatogram for base-edited hiPSC cells and MYH7 expression analysis. Sanger sequencing analysis showed a single chromatogram peak at the targeted genomic positions, with no detectable off-target modifications. (A) hiPSC clone showing the presence of both C and T for the *MYH6* gene (R725C: heterozygous). (B) hiPSC clone showing C to T conversion for the *MYH7* gene (R723C: homozygous). (C) Brightfield images of isogenic control hiPSC and *MYH6/7* mutant hiPSC cells at 20X magnification. Scale bar: 20μm. (D) The relative expression levels of *MYH7* transcripts, analyzed by qPCR at days 20, 30, and 45. n=6 (Each dot represents an individual replicate from three independent biological replicates in duplicates). Data are presented as mean ± SD. Statistical significance and the *p-value* between the isogenic control and *MYH6/7* mutant was determined by using an *unpaired Welch's t-test*.

Supplemental Figure S2: Contractility assessment in *MYH6/7* mutant CMs. (A, B) Max amplitude (A) and peak time (B) were calculated for the isogenic control and the *MYH6/7* mutant at day 20, day 30, and day 45. Each data point represents the individual technical replicate from n=3 independent biological replicates. (n=9) (Each dot represents a technical replicate from n=3 independent biological replicates). Data are presented as mean ± SD. Statistical significance and the *p-value* between the isogenic control and *MYH6/7* mutant was determined by using *unpaired Welch's t-test*.

Supplemental Figure S3: *MYH6/7* mutations alter nuclear morphology in hiPSC-CMs. (A-B) Nuclear morphology analysis showed that there is no significant difference in the nuclear area (A) between the isogenic control and *MYH6/7* mutant. Nuclear aspect ratio (B) significantly increases at day 45 but remains unchanged at other time points. Analysis was done using ~150-300 hiPSC-CMs per condition, obtained from n=3 independent biological replicates. Data are presented as mean ± SD. Statistical significance and the *p-value* between the isogenic control and *MYH6/7* mutant was determined by using *unpaired Welch's t-test*. (C-E) The frequency distribution histogram for the nuclear area of each cell did not show any significant change in the *MYH6/7* mutant when compared to the isogenic control at day 20 (*p=0.55*), day 30 (*p=0.22*), or day 45

(*p=0.44*). Analysis was done using ~100 hiPSC-CMs per condition, obtained from n=3 independent biological replicates. Data are presented as mean ± SD. Statistical significance and the *p-value* between the isogenic control and *MYH6/7* mutant was determined by using *unpaired Welch's t-test*.

Supplemental Figure S4: qPCR analysis of Ca^{2+} handling transcripts in control and mutant hiPSC-CMs. (A-C) Expression levels of physiological functions transcripts *GJA1* (A)*, KCNJ2* (B)*,* and *RYR2* (C) were analyzed by qPCR at day 20, day 30, and day 45. n=6 (Each dot represents an individual technical replicate from three independent biological replicates). Data are presented as mean ± SD. Statistical significance and the *p-value* between the isogenic control and *MYH6/7* mutant was determined by using *unpaired Welch's t-test*.

Supplementary Table 1: List of qPCR primers used in this study.

Supplementary Table 2: List of antibodies and staining reagents used in this study.

Supplemental Figure S1

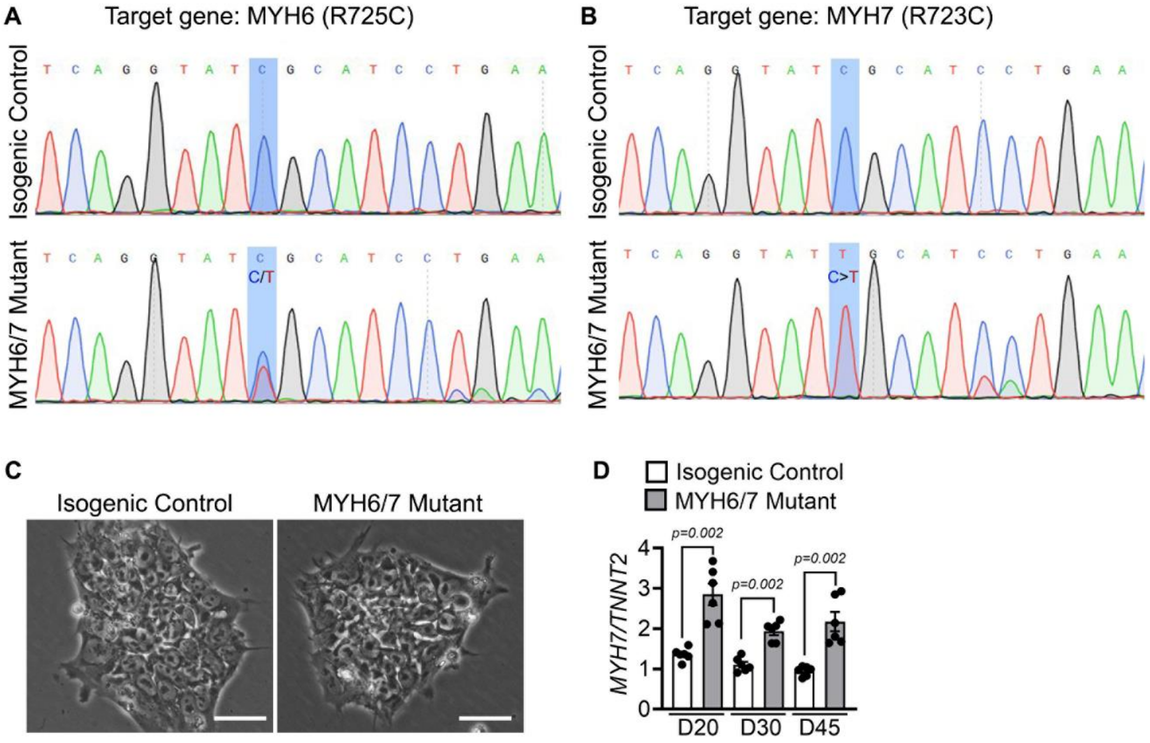

Supplemental Figure S2

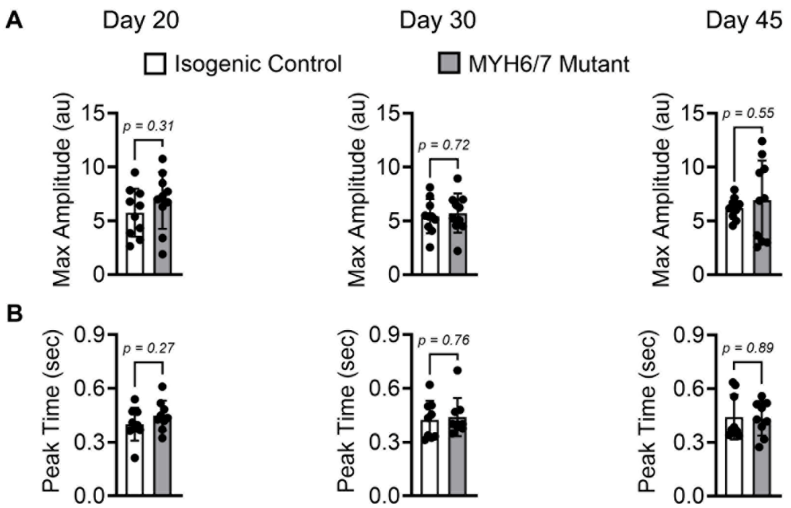

Supplemental Figure S3

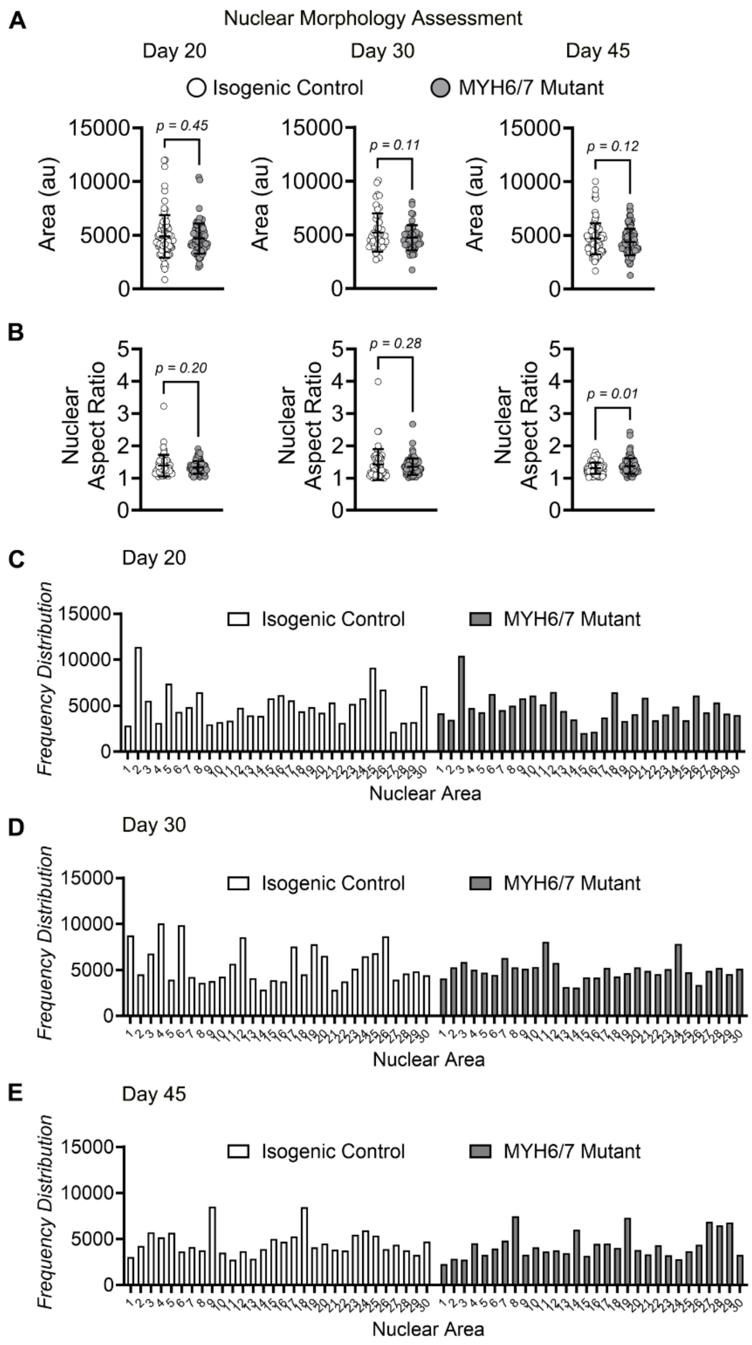

Supplemental Figure S4

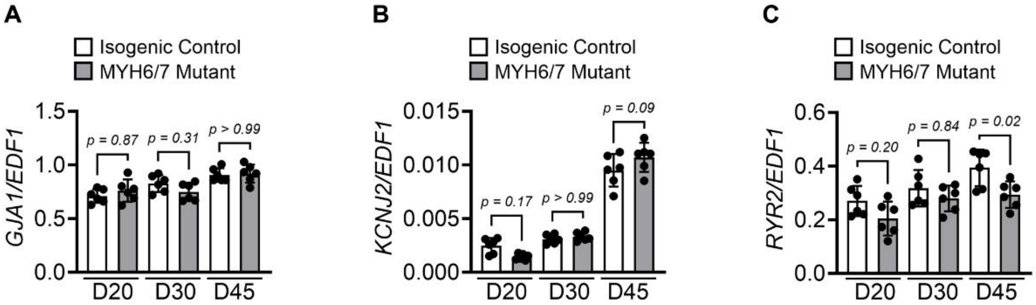

Supplemental Table S1: qPCR Primer Sequence

	Gene	Forward (5'- 3')	Reverse (5'- 3')
1	***Endothelial differentiation related factor 1 (EDF1)***		
		ATCTTAGCGGCACAGAGACGAG	TGTCATGGTGCAGCTCCTCTGT
2	***Actin-α cardiac muscle 1 (ACTC1)***		
		CTGGCTCCTAGCACCATGAAG	TGCGGTGGACAATGGATGG
3	***α-Actinin-1(ACTN1)***		
		AGGACCGTGTGGAGCAGATTG	CAGATTGTCCCACTGGTCACA
4	***Dystrobrevin-α (DTNA)***		
		CGGCTTGATGAAGAACACAGG	AGATGTCAGGAGCACTTCTCTG
5	***α-myosin heavy chain (MYH6)***		
		GGAAGACAAGGTCAACAGCCTGT	TCCAGTTTCCGCTTTGCTCGC
6	***Gap junction alpha-1 (GJA1)***		
		GGAGATGAGCAGTCTGCCTTTCG	ATGAGCCAGGTACAAGAGTGTGG
7	***Potassium Inwardly Rectifying Channel Subfamily J Member 2 (KCNJ2)***		
		AACAGTGCAGGAGCCGCTTTGT	AGGACGAAAGCCAGGCAGAAGA
8	***Ryanodine receptor 2 (RYR2)***		
		TCTTGAGGTTGGCTTTCTGCCAG	CTGTGCCAGCAAAGAGAGGAGCA
9	***Natriuretic peptide A (NPPA)***		
		TACAATGCCGTGTCCAACGCAG	CTTCATTCGGCTCACTGAGCACT
10	***Natriuretic peptide B (NPPB)***		
		GGCACCGCAAAATGGTCCTCT	AGCCAGACCCTTGCACCATCTTG
11	***Four-and-a-half LIM domains 1 (FHL1)***		
		GTTTCACCGCTGTGGAGGAC	GTGGAAGCAGTAGTCGTGCC
12	***Four-and-a-half LIM domains 2 (FHL2)***		
		TGGTGTGCTTTGAGACCCTG	AGCAGTGGAAACAGGCTTCATG

Supplemental Table S2: Antibodies used in the study.

Antibody	Manufacturer	Catalog #	Antibody dilution
Anti-Sarcomeric α-actinin antibody [EA-53]	Abcam	ab9465	1:500
Donkey anti-Mouse IgG Alexa Fluor 488	Thermo fisher Scientific	A21202	1:400
Rhodamine Phalloidin	Abcam	AB235138	1:1000
DAPI (4',6-Diamidino-2-Phenylindole)	Thermo fisher Scientific	D1306	1:1000
